# Rotating magnetic field delays human umbilical vein endothelial cell aging and prolongs the lifespan of *Caenorhabditis elegans*

**DOI:** 10.18632/aging.102466

**Published:** 2019-11-22

**Authors:** Jiangyao Xu, Kan Liu, Tingting Chen, Tianying Zhan, Zijun Ouyang, Yushu Wang, Wen Liu, Xiaoyun Zhang, Yang Sun, Gaixia Xu, Xiaomei Wang

**Affiliations:** 1 Base for International Science and Technology Cooperation: Carson Cancer Stem Cell Vaccines R&D Center, Shenzhen Key Lab of Synthetic Biology, Department of Physiology, School of Basic Medical Sciences Shenzhen University, Shenzhen 518055, China; 2 Guangdong Key Laboratory for Biomedical Measurements and Ultrasound Imaging, School of Biomedical Engineering, Shenzhen University, Shenzhen 518055, China; 3 State Key Laboratory of Pharmaceutical Biotechnology, Department of Biotechnology and Pharmaceutical Sciences, School of Life Sciences, Nanjing University, Nanjing 210023, China

**Keywords:** RMF, C. elegans, HUVEC, IIS, senescence

## Abstract

The biological effects of magnetic fields are a research hotspot in the field of biomedical engineering. In this study, we further investigated the effects of a rotating magnetic field (RMF; 0.2 T, 4 Hz) on the growth of human umbilical vein endothelial cells (HUVECs) and *Caenorhabditis elegans*. The results showed that RMF exposure prolonged the lifespan of *C. elegans* and slowed the aging of HUVECs. RMF treatment of HUVECs showed that activation of adenosine 5'-monophosphate (AMP)-activated protein kinase (AMPK) was associated with decreased mitochondrial membrane potential (MMP) due to increased intracellular Ca^2+^ concentrations induced by endoplasmic reticulum stress in anti-aging mechanisms. RMF also promoted the health status of *C. elegans* by improving activity, reducing age-related pigment accumulation, delaying Aβ-induced paralysis and increasing resistance to heat and oxidative stress. The prolonged lifespan of *C. elegans* was associated with decreased levels of daf-16 which related to the insulin/insulin-like growth factor signaling pathway (IIS) activity and reactive oxygen species (ROS), whereas the heat shock transcription factor-1 (hsf-1) pathway was not involved. Moreover, the level of autophagy was increased after RMF treatment. These findings expand our understanding of the potential mechanisms by which RMF treatment prolongs lifespan.

## INTRODUCTION

The biological effects of exposure to magnetic fields have been widely debated [1–3]. Furthermore, the effects magnetic fields on cell function and metabolism have been extensively investigated in many areas such as cancer therapy [4], bone development [5], and electrophysiology [6]. Magnetic fields can be divided into static and dynamic fields, with the latter further divided into sinusoidal alternating, pulse electromagnetic, pulsating electric and rotating magnetic fields. Because the direction of the rotating magnetic field (RMF) changes constantly with the rotation of the magnet, it has certain representativeness in the study of the biological effect of magnetic field [7].

The changes in protein structure induced by magnetic fields and the electrophysiological function of magnetic fields are the most controversial topics in biophysics [8–11]. Many species utilize the geomagnetic field for navigation, long-distance migration, and avoidance of natural disasters [12–15]. Although several animal organs or cells containing magnetic nano-particles have been shown, including pigeons and whales, these have either been shown to be unrelated to magnetic reception, or the results lack validation [16, 17]. In 2016, the Xie group published the first report of a hypothetical magnetic receptor (MagR) with a unique structure consisting of a multimeric magnetic induction rod-like protein complex. [18] however, the underlying mechanism by which magnetic fields influence life is unclear [19].

Several hypotheses have been suggested to explain the biological effects of magnetic fields. The eddy current hypothesis of rotating magnetic fields (RMF) was pioneered by Hubley [20]. Calcium ions (Ca^2+^), a class of second messenger closely related to metabolism, remains the best biochemical magneto-effector candidate [21, 22]. The endoplasmic reticulum functions as a storage site for intracellular Ca^2+^. Under conditions of endoplasmic reticulum stress, Ca^2+^ is released into the cytoplasm, resulting in increased intracellular Ca^2+^ concentration. It has been reported that the endoplasmic reticulum “perceives” magnetic information by the magnetic spin dynamics of the Ca^2+^ protein channel reaction induced by eddy currents [23, 24]. Identification of inhibitory osteoblasts exhibiting magnetic induction behavior within the endoplasmic reticulum represent the first practical experimental evidence that Ca^2+^ is required for magnetic induction pathways in bone development [25, 26]. The response of the endoplasmic reticulum to the magnetic field through the Ca^2+^ channel can be used to perceive biophysical information from the magnetic field. The endoplasmic reticulum has been proposed as an enabler of magnetic field intensity-and direction-guided behavior in animals via mechanism that is consistent with an electron eddy hypothesis [27–29]. This hypothesis is based on a requirement for magnetic minerals, such as iron, and proteins containing magnetic substances that act as magnetobiological receptors, which receive and respond to magnetic changes. However, the identification and isolation of such ferromagnetic proteins in the endoplasmic reticulum has been difficult. In addition, changes in mitochondrial membrane potential (MMP) as well as cellular Ca^2+^ concentrations have been reported under the influence of magnetic fields [30–34].

As a replicative senescent cell type, human umbilical vein endothelial cells (HUVECs) express a series of markers of senescence and angiogenesis, which can be used as an indicator of cell aging [35–37]. Glucose is a useful agent for accelerating aging *in vitro* and facilitates exploration of the underlying molecular events [38–40]. In this study, we used an *in vitro* model of senescence using high glucose-stimulated HUVECs to examine the molecular events underlying the aging process. Many previous studies have shown that anti-oxidases promote anti-aging effects and promote cellular proliferation [41–43].

*Caenorhabditis elegans* (*C. elegans*) is an independent nematode that has been widely used as an animal model for studying aging and neurodegenerative disorders such as Alzheimer’s disease and Parkinson’s diseases [44]. Compared with traditional animal models, this small nematode has a series of advantages, such as small size, short lifespan, availability of the complete genome sequence, over 65% genes related to human diseases, and a transparent body, which is conducive to the observation of fluorescent marker expression [45–48]. All of these characteristics make this organism as an ideal system for studying the biological effects of magnetic fields *in vivo* [49–54].

The magnetite-based electron eddy hypothesis has credible theoretical and experimental foundations. Magnetic induction behavior is very common in animals; some animals and plants can sense the energy, frequency, intensity and direction of the magnetic field via a single magnetic induction receptor. Therefore, it is possible to form a magnetic induction pathway consisting of a plurality of magnetic field sensing proteins.

In this study, we aimed to verify the function of the Ca^2+^ channel in the endoplasmic reticulum as a magnetic field-induced receptor. Combining physics with biology, the results of our study are very important in highlighting a range of MF applications in the future.

## RESULTS

### RMF characterization

**Figure 1:**
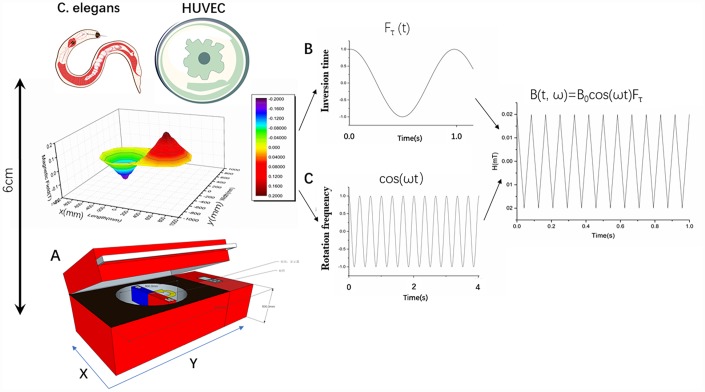
**Characterization of rotating magnetic field and experimental setup.** (**A**) Experimental setup for treatment of *Caenorhabditis elegans* and cells with a rotating magnetic field (RMF). *C. elegans* and cells were positioned above the RMF generator and exposed to RMFs of different amplitudes consisting of two overlaying components: translational (with varying inversion time) and rotational (with varying rotational frequencies). (**B**, **C**) B(t, ω) represents the magnetic field induction as a function of time; B0 represents the amplitude; Fτ represents the contribution of the translational movement of different inversion time; and ωt represents the contributing rotation frequency.

The entire experimental equipment consisted mainly of natural magnets (Figure 1A). The experimental material was placed 6 cm (0.2 T) from the motor-driven rotating magnet, which produced a 4 Hz magnetic field change frequency. The frequency of the magnet rotation and the timing of the changes in the magnetic field direction were constant and the entire experimental setup provided an environment for culturing the cells (Figure 1B, 1C).

### RMF delayed HUVEC aging

**Figure 2:**
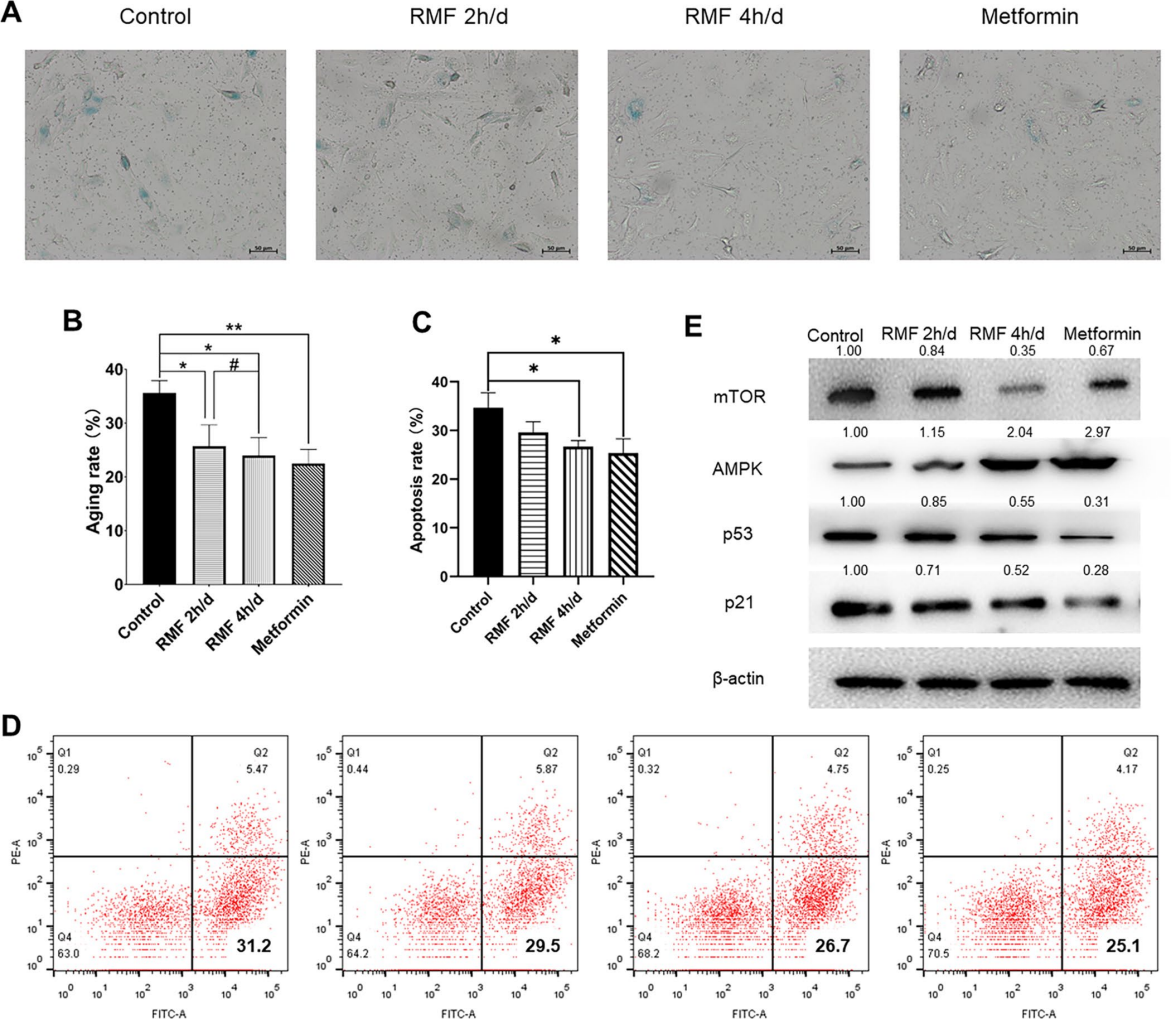
**Rotating magnetic field exposure delays HUVEC senescence.** (**A**, **B**) HUVECs were exposed to RMF daily for 0 h, 2 h, and 4 h; 20 μM metformin was used as a positive control. SA-β-Gal staining was performed and the number of β-Gal-positive (blue) cells was calculated as a percentage of the total cell number using Image J software. (**C**, **D**) Flow cytometry was used to detect the apoptosis of HUVEC after RMF treatment. (**E**) Western blot analysis showed that RMF exposure increased in AMPK protein expression and decreased P21, P53 and mTOR protein expression.

In the logarithmic growth phase of cells, HUVECs were cultured in high-glucose (40 mM) to construct a cell senescence model. As a classic anti-aging drug, metformin (20 μM) was used as a positive control. HUVECs were exposed to RMF for 2 h/d and 4 h/d and cell senescence was measured after 3 d. The number of SA-β-Gal-positive cells decreased with increasing RMF exposure time (Figure 2A, 2B. *P*<0.01), and flow cytometric analysis revealed that the number of apoptotic cells was decreased (Figure 2C, 2D. *P*<0.01). Western blot analysis showed that RMF exposure increased AMPK protein expression, and decreased expression of P21, P53 and mTOR proteins in a time-dependent manner (Figure 2E). The results indicated that RMF delays the senescence of HUVECs by upregulating signaling via the AMPK pathway and downregulating the mTOR pathway.

### HUVEC genome sequencing

**Figure 3:**
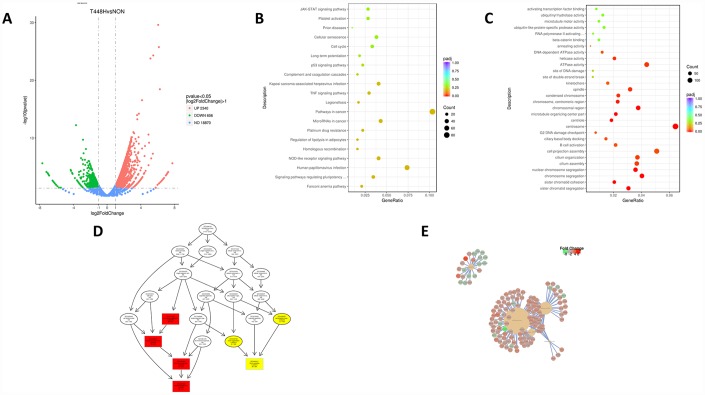
**RMF extends the life of HUVECs in a multi-target manner.** (**A**) Volcano map showing the RNA-seq expression pattern in HUVECs exposed to RMF for 4 h compared to that of untreated control cells. Red indicates a higher expression level and green indicates a lower expression level. The distance of the dot from the x-axis reflects the size of the *P*-value. (**B**, **C**) Gene enrichment map in HUVECs exposed to a RMF for 4 h compared to untreated control cells. (**D**) Signal transduction pathways affected by RMF. (**E**) Predicted protein interactions.

To broadly identify the mechanisms underlying RMF-induced longevity, we performed RNA sequencing analysis of HUVECs with or without exposure to RMF for 4 h. Transcriptome volcano map analysis showed that, after 4 h of RMF treatment, 2,340 gene were upregulated genes and 656 downregulated genes (Figure 3A). GO and KEGG analysis indicated that the downregulated genes were mainly involved in centrosomes, cell projection assembly, and ATP activity, suggesting that RMF adjusted the energy metabolism of HUVECs (Figure 3B, 3C). In addition, we speculated on the possible protein pathways and interactions (Figure 3D, 3E). The results showed that RMF influences the senescence of HUVECs by affecting the activity of ATPase activation, centrosomes, and cell projection assembly.

### RMF improved Ca^2+^ channel permeability in the endoplasmic reticulum and decreases ATP production

**Figure 4:**
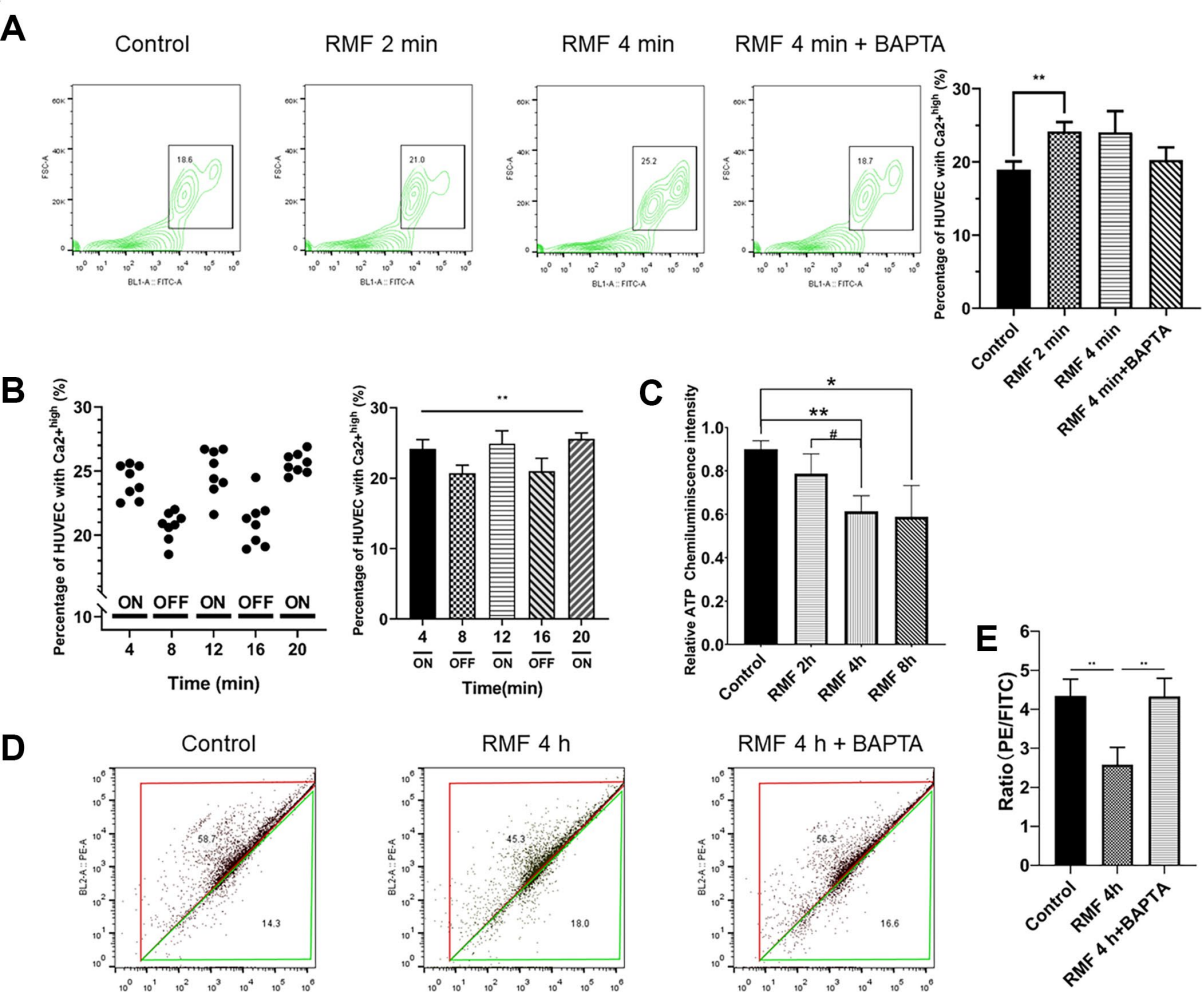
**RMF induced Ca^2+^ outflow from the endoplasmic reticulum to reduce intracellular MMP and ATP levels**. (**A**) HUVECs were treated with RMF for 0 min, 2 min, 4 min and 4 min with a calcium channel inhibitor (BAPTA). After the addition of the Ca^2+^ probe Fluo-4-AM, the intracellular Ca^2+^ concentration was detected by flow cytometry. (**B**) The intracellular Ca^2+^ concentration in the same cells was detected following RMF treatment at 4-min intervals. (**C**) HUVECs were treated with RMF for 0 h, 2 h, 4 h, and 8 h. The intracellular ATP content was then detected. (**D**, **E**) After RMF treatment of HUVECs at 0 min, and 4 min in the presence and absence of the endoplasmic reticulum calcium channel inhibitor (BAPTA), changes in MMP levels were detected by flow cytometric analysis of the MMP dye, JC-1.

To further explore the mechanism of RMF resistance to endothelial cell senescence, we examined the levels of Ca^2+^ and ATP. The results showed that the concentration of Ca^2+^ was obviously increased in glucose-induced senescent HUVECs after RMF treatment. The intracellular Ca^2+^ content changed very fast due to magnetic field interference, and the increase in Ca^2+^ concentration slowed as the RMF treatment time increased (Figure 4A. *P*<0.01). There was no significant difference between the effects of exposure to RMF for 4 min and 2 min, indicating that the RMF-induced increase in intracellular Ca^2+^ concentration in HUVECs reached saturation at a rapid rate within 4 min. In this study, we used the endoplasmic reticulum calcium channel-specific inhibitor, BAPTA (25 μM). There was no significant increase in Ca^2+^ content in the inhibitor-treated group compared to that in the control group, indicating that the calcium channel in the endoplasmic reticulum is a magnetic field target. Subsequently, changes in Ca^2+^ concentration in the same batch of HUVECs were detected by continuously turning the magnetic field on and off at intervals of 4 min (Figure 4B. *P>*0.01). When the RMF was turned on, the intracellular Ca^2+^ concentration increased, whereas when the RMF was turned off, the intracellular Ca^2+^ concentration slowly decreased, which provides evidence that the calcium ion channel in the endoplasmic reticulum is a sensitive target of the magnetic field. Thereafter, we examined changes in the MMP by flow cytometric analysis of JC1-stained HUVECs. The results showed that as the intracellular Ca^2+^ concentration increased (Figure 4D, E. *P*<0.01), the MMP difference and intracellular ATP production decreased (Figure 4C. *P*<0.01). These findings indicated that RMF reduces mitochondrial activity by increasing the intracellular Ca^2+^ concentration.

### RMF increased the lifespan of *C. elegans* and reduced damage under stress conditions

**Figure 5:**
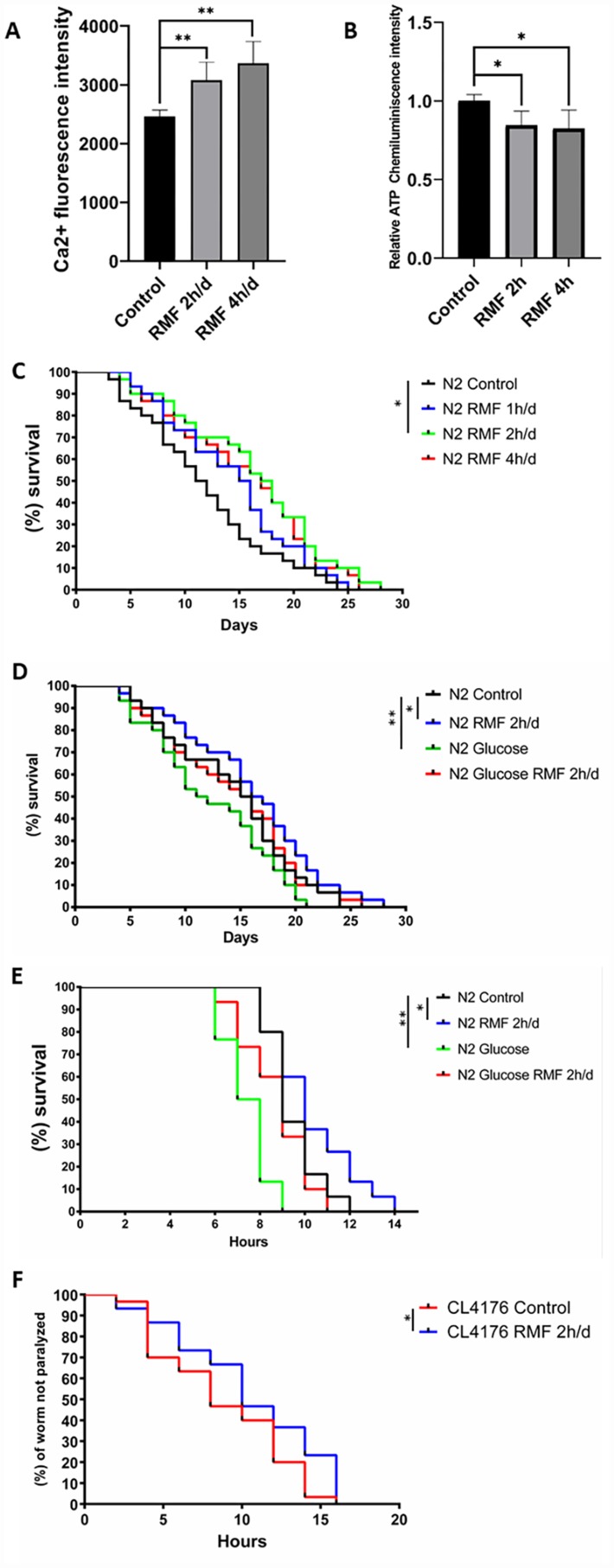
**RMF increased the lifespan of *C. elegans* and reduced damage under stress conditions.** (**A**, **B**) Changes of Ca2+ and ATP levels in *C. elegans* after RMF treatment. (**C**) The lifespan of 240 N2 *C. elegans* eggs following RMF treatment for 0 h/d (control), 1 h/d, 2 h/d and 4 h/d was recorded. (**D**) The lifespan of 240 N2 *C. elegans* eggs following RMF treatment for 0 h/d (control) and 2 h/d in the presence and absence of glucose was recorded. (**E**) Worms were treated with RMF for 0 h/d (control) and 2 h/d at 20°C for 4 days, and then exposed to thermal shock at 37°C. (**F**) Curves showing the non-paralyzed fraction nematodes in each group following treatment of *C. elegans* eggs with RMF for 0 h/d (control) and 2 h/d. CL4176 *C. elegans* were raised from the egg stage to the L3 stage at 15°C and then transferred to an environment at 25°C.

We found that the levels of Ca2+ and ATP in the *C. elegans* exposed to RMF were consistent with HUVECs (Figure 5A, 5B). Lifespan is the most intuitive indicator to study the effects of magnetic fields on Aging evaluative systems. Because of its short growth cycle, ease to observation and high degree of gene homology with humans, *C. elegans* is an ideal model organism to study the effect of RMF on lifespan. In this study, exposure of *C. elegans* to RMF at 1 h/d, 2 h/d and 4 h/d revealed that RMF significantly increased the average lifespan by 0.6%, 13.99% and 8.39%, respectively, compared with the control (Table 1), with the most significant effect observed following 2 h/d RMF exposure (Figure 5C, Table 1. *P*<0.01); therefore, 2 h/d RMF was used for subsequent experiments.

**Table 1:** Statistical analysis of the lifespan of *C. elegans*.

**Condition**	**Treatment**	**Mean lifespan**	**Median lifespan**	**Maximum lifespan**	**Mean fold change**
N2 (20°C, days)	Control	14.3±0.32	15.5±0.69	24±0.66	—
	Glucose	12.3±0.58	11.5±0.68	21±0.79	-13.99%
	RMF 1h/d	14.4±0.36	15.5±1.14	25±0.34	0.60%
	RMF 2h/d	16.3±0.54	17.5±0.78	28±1.23	13.99%
	RMF 4h/d	15.5±1.12	17.0±0.81	26±0.94	8.39%
	Glucose RMF 2h/d	14.1±0.69	15.5±0.69	26±0.64	0.98%
N2 (37°C, hours)	Control	9.4 ±1.23	9.0 ±0.31	12±0.28	—
	RMF 2h/d	10.2±0.97	10.0±0.77	14±0.24	8.51%
	Glucose	7.4 ±0.64	7.5 ±0.46	9 ±0.61	-21.28%
	Glucose RMF 2h/d	8.7 ±0.58	9.0 ±1.27	11±0.87	-7.45%

The data were analyzed by one-way ANOVA (SPSS 18), and different letters in a column denote values that are significantly different (*P* < 0.05). The mean fold increase was calculated by (T-C)/C×100, where T is the mean survival time of *C. elegans* treated with RMF and C is the mean survive time of the control.

Glucose causes developmental retardation and shortens the lifespan of *C. elegans*; therefore, we investigated the effect of RMF on the longevity of *C. elegans* exposed to toxic glucose stimuli. We observed that RMF attenuated the toxic effects of glucose on *C. elegans*, with the average life increased by 14.63% (Figure 5D, Table 1. *P*<0.01) compared with that of the control.

The resistance of *C. elegans* to external stimuli decreases dramatically with age. To determine the ability of RMF to increase the resistance of *C. elegans* to heat stress, we analyzed the survival of day 7 *C. elegans* treated with 2 h/d RMF under heat stress conditions at 37°C. We found that RMF preconditioning had a significant protective effect against this acute stress (Figure 5E. *P*<0.01), indicating that RMF increased the resistance of *C. elegans* to heat stress. Increased longevity has been reported to be closely correlated with the prevention of age-related diseases. Extensive experimental studies have shown that accumulation of β-amyloid (Aβ) in Alzheimer’s disease is a core event that triggers neuronal degeneration. In CL4176 *C. elegans*, the smg-1 system is inactivated by raising the temperature, resulting in high Aβ 1–42 expression in muscle tissue and formation of a polymer. The mutant CL4176 cultured under RMF showed a significant anti-disease effect compared to that observed in the control group. The average lifespan of CL4176 was extended by 19.32% (Figure 5F, Table 2. *P*<0.05). These results showed that RMF increases the lifespan of *C. elegans* and reduces the damage under stress conditions.

**Table 2:** Statistical analysis of paralysis of *C. elegans*.

**Condition**	**Treatment**	**Mean**	**Median**	**Maximum**	**Mean fold increase**
CL4176(25°C, quantity)	Control	8.8±1.03	8.0±0.39	16±1.09	—
	RMF 2h/d	10.5±0.71	10.0±0.73	16±0.91	19.32%

The data were analyzed by one-way ANOVA (SPSS 18), and different letters in a column denote values that are significantly different (*P* < 0.05). The mean fold increase was calculated by (T-C)/C×100, where T is the mean paralysis time of *C. elegans* treated with RMF and C is the mean paralysis time of the control.

### RMF improved *C. elegans* healthspan

**Figure 6:**
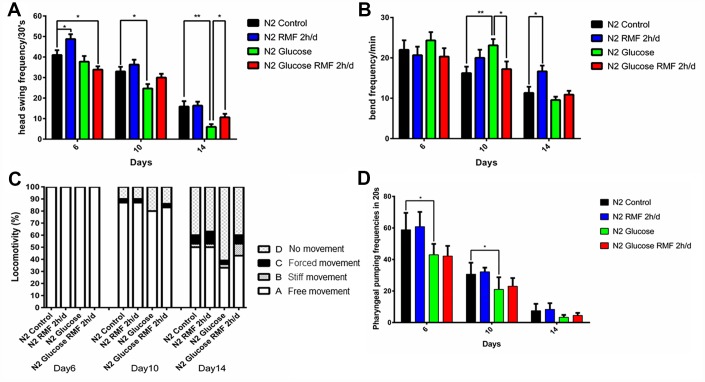
**RMF improved lifespan in *C. elegans*.** (**A**) Head-swing frequency. (**B**) Body-bend frequency. (**C**) The four levels of locomotivity. Motor ability assays were performed on day 6, 10, and 14. (**D**) Pharyngeal-pumping frequency (20 s). Different letters indicate a significant difference among groups (*P* < 0.05). All analyses were based on the data from three independent experiments.

**Figure 7:**
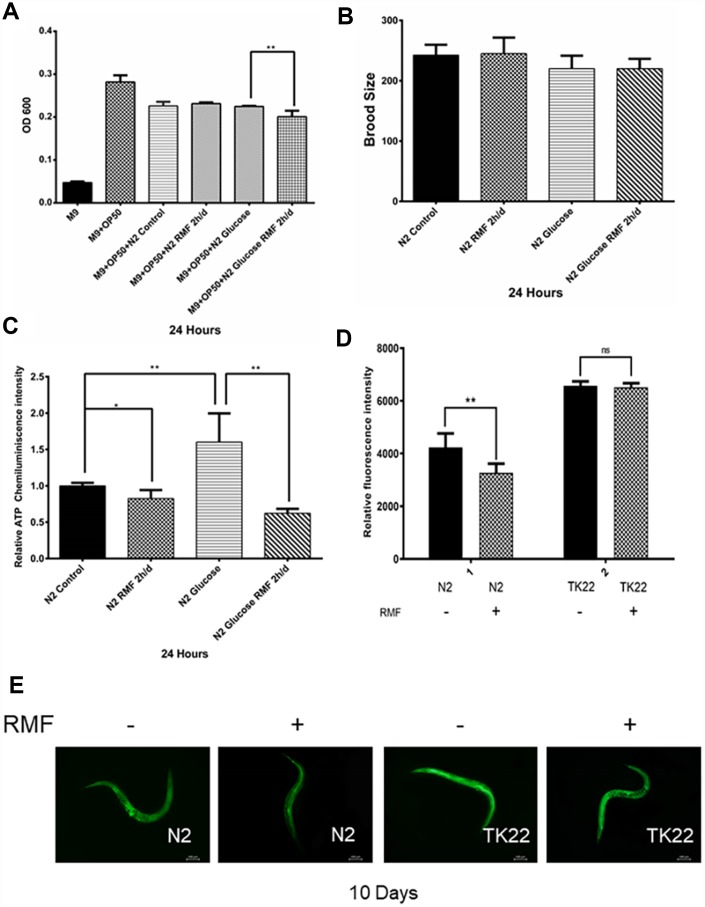
**The effects of RMF on ROS and ATP levels and *C. elegans* physiology.** (**A**) *C. elegans* food-intake. (**B**) *C. elegans* brood size. (**C**) Total ATP content in *C. elegans* was detected using a microplate reader. (**D**, **E**) The ROS levels in *C. elegans* were detected with the H2DCF–DA probe by fluorescence microscope and microplate reader.

Healthspan is a new term for the period during which adult activity and function remain intact prior to age-related decline. As the incidence of age-related diseases increases, the impact of current life-extending methods on health is unclear. To determine whether RMF simply extends life or merely youth and health, we assessed the effects of RMF on aging-related physiological functions, including head-swinging, body-bending, food-intake, and distance moved. During aging, muscle cells gradually lose their vitality, leading to decreased exercise ability and pharyngeal pump function as well as changes in other phenotypes. We measured pharyngeal pump volume and body curvature in RMF-treated animals on days 6, 10, and 14, respectively, as indicators of physiological changes. RMF significantly increased the flexibility of body-bending and the frequency of pharyngeal muscle contraction (Figure 6A–6D. *P*<0.01). Furthermore, RMF had a slight but not significant increase in N2 *C. elegans* food-intake measured in day 10, suggesting that its effect on longevity may not be dependent on the dietary restriction (DR) pathway (Figure 7A. *P*>0.05). Interestingly, food-intake increased significantly in RMF-treated *C. elegans* under high-glucose conditions, indicating the involvement of other mechanisms that remain to be explored. Previous studies have shown that gonadal function declines with age. Prolonging the lifespan of *C. elegans* may have reproductive side-effects. RMF treatment had no effect on brood size, suggesting that RMF-induced longevity was not dependent on reproductive signaling pathways (Figure 7B. *P*>0.05). These results showed that RMF exposure significantly prolonged the youthful vigor and healthy life of *C. elegans*. Subsequently, we showed that the decrease in ATP levels observed in RMF-treated *C. elegans* reflected that in HUVEC (Figure 7C. *P*<0.01).

### RMF reduced ROS levels in *C. elegans* via a mev-1-dependent pathway

In addition to the ROS produced during metabolism, the endogenous ROS levels are increased by adverse external stimuli accelerate the aging process. Furthermore, studies have shown that *C. elegans* produces large amounts of ROS in high-glucose environments [55]. To investigate the ability of RMF to promote antioxidant mechanisms, including clearance of ROS, we used H2DCF-DA as a free radical probe to compare ROS levels in *C. elegans* exposed to RMF for 10 days and those in the control group. The results showed that RMF pretreatment had a significant protective effect against the increased ROS production induced by a high-glucose stress environment, indicating that the increased lifespan induced by RMF treatment was related to reduced ROS accumulation and enhanced resistance of *C. elegans* to oxidation (Figure 7D, 7E, *P*<0.01). Studies have shown that the *mev-1* gene affects the expression of antioxidant enzymes. The mev-1 mutant *C. elegans* carries a defective complex II subunit in the electron transport chain (ETC), which significantly shortens life at high oxygen concentrations. Thus, we investigated the possible antioxidant effects of RMF on TK22 *C. elegans*, which carries a mutation in the mitochondrial complex II cytochrome B large subunit. There was no significant difference in ROS levels between the two *mev-1*-mutation carrying forms of *C. elegans*, suggesting that the *mev-1* gene is critically involved in the mechanism by which RMF extends life.

### RMF reduced the accumulation of fat and age-related pigments in *C. elegans*

**Figure 8:**
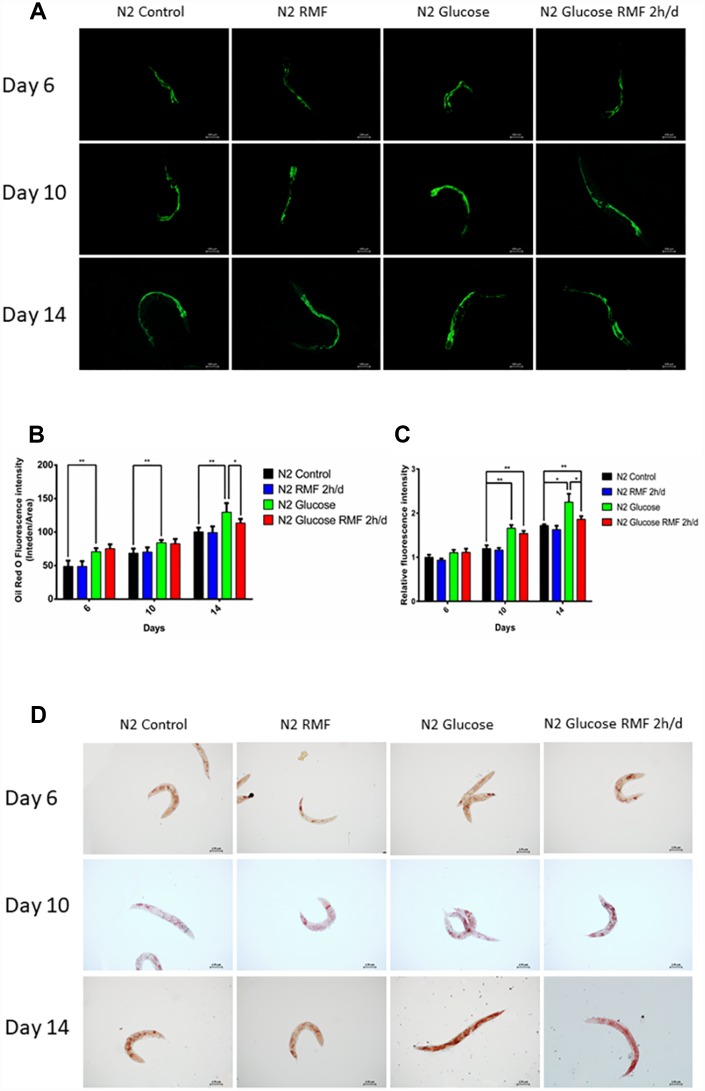
**RMF reduced the accumulation of fat and age-related pigments in *C. elegans*.** (**A**) Representative image of age-related pigments in N2 *C. elegans*; scale bar = 100 μm. (**B**) The fluorescence of the fat normalized to tryptophan fluorescence was measured using a microplate reader. (**C**) The fluorescence of the age-related pigment normalized to tryptophan fluorescence was measured using a microplate reader. (**D**) Representative image of fat in N2 *C. elegans*; scale bar = 100 μm. Both experiments were performed on four independent four occasions (days 6, 10 and 14 after the start of RMF treatment). Different letters indicate a significant difference among groups (*P* < 0.05).

Age-related pigments are a biomarker of aging of *C. elegans* and humans*.* In this study, we employed a fluoroenzyme marker to show that the fluorescence intensity of age-related pigments was significantly reduced in *C. elegans* on day 10 and 14 after RMF treatment (Figure 8A, 8B. P<0.01).

In *C. elegans*, an increase in ROS production leads to a marked fat accumulation. Considering our previous observation that RMF effectively reduced ROS accumulation, we next investigated the effects of RMF and glucose pretreatment on fat accumulation in N2 *C. elegans*. At day 6, 10, and 14 after RMF treatment, by oil red O staining showed that fat accumulation was reduced by 2 h/d RMF compared with the control (Figure 8C, 8D. *P*<0.01).

### RMF improved autophagy

**Figure 9:**
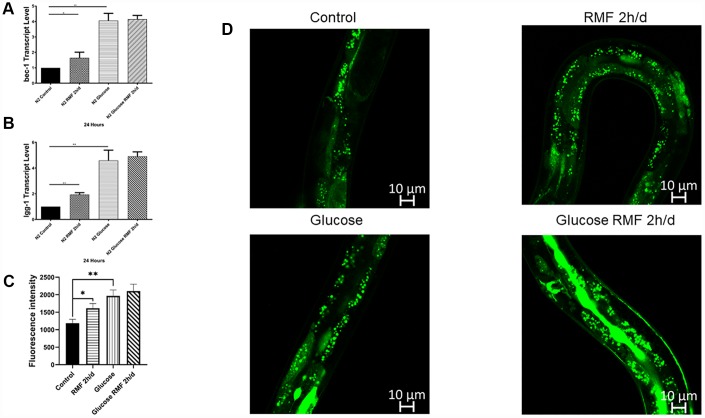
**Autophagy was involved in lifespan extension and protection against Aβ-induced toxicity by RMF.** (**A**, **B**) The *lgg-1* and *bec-1* gene transcript levels in CL4176 *C. elegans*. (**C**, **D**) dFP::LGG-1 was detected by laser confocal microscope (Leica-sp5ii, TCS SP5II, Shenzhen).

Autophagy is the phagocytosis of cytoplasmic proteins or organelles in a process that occurs in vesicles, which fuse with lysosomes to form autophagosomes. The contents of these organelles are then degraded, thereby realizing the metabolic needs of cells and the renewal of some organelles. Numerous studies have demonstrated that abnormalities in autophagy function are closely related to the development of age-related diseases such as Alzheimer’s disease. Bec-1 is a key positive regulator of the level of autophagy. LGG, which is the homolog of mammalian LC3, is one of the well- described and widely available of the autophagy matrices and is used as a marker for autophagy measurement because of its involvement in the formation of autophagic vacuoles. In this study, we found that *bec-1* mRNA levels were increased 2-fold compared with those in the control group after 10 days of 2 h/d RMF treatment, (Figure 9A), and LGG mRNA levels were increased 2.5-fold compared with those in the control group (Figure 9B, *P*<0.01). The effect of RMF on autophagy activity was assessed in the DLM1 strain, which expresses the LGG-1-GFP fusion protein *in vivo*. The results showed that both RMF and glucose treatment increased autophagy in *C. elegans* (Figure 9C, 9D, *P*<0.01). The relationship between autophagy and aging is controversial; however, our studies showed that RMF induces an appropriate autophagy pathway to prolong life and reduce Aβ-induced toxic effects in *C. elegans*.

### RMF prolonged the lifespan of *C. elegans* via the insulin/IGF-1 signaling pathway

**Figure 10:**
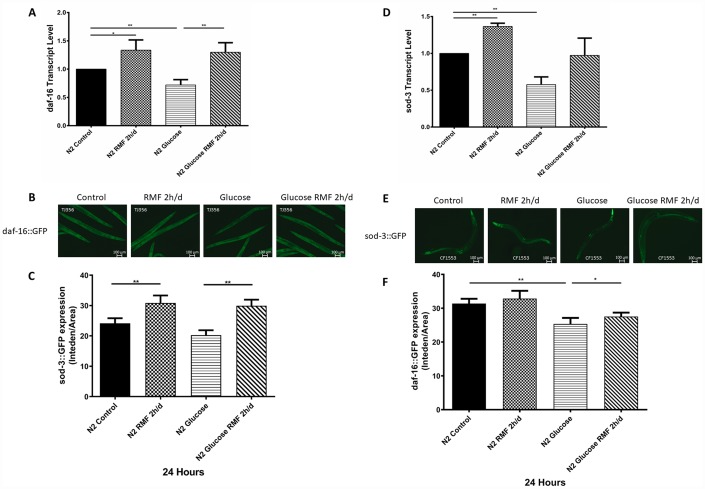
**RMF promoted the expression of daf-16 and SOD-3.** (**A**) The daf-16 transcript level. (**B**) Expression of daf-16::GFP observed under a fluorescence microscope (**C**) The relative fluorescence intensity of the daf-16::GFP was quantified using ImageJ software. (**D**) The SOD-3 transcript level. (**E**) Expression of SOD-3::GFP expression observed under a fluorescence microscope. (**F**) The relative fluorescence intensity of the SOD-3::GFP was quantified using ImageJ software.

**Figure 11:**
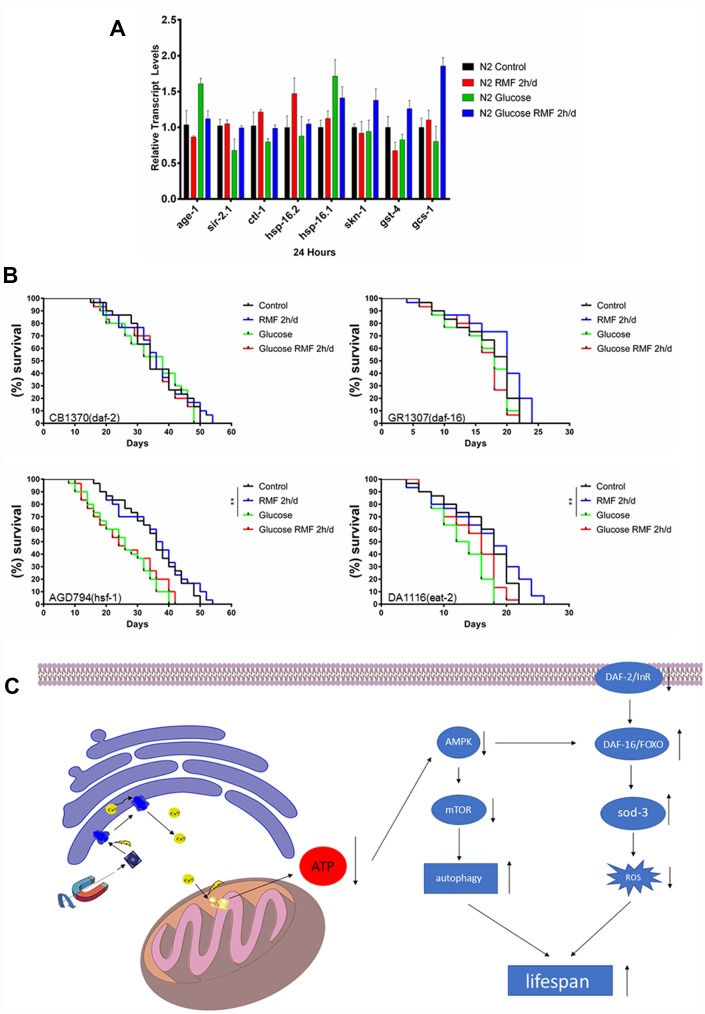
**The molecular mechanism underlying the anti-aging effect of RMF.** (**A**) The mRNA levels of *hsp-16.1*, *hsp-16.2*, *age-1*, *sir-2.1*, *ctl-1*, *skn-1*, *gst-4* and *gcs-1* were determined by quantitative real-time RT-PCR and normalized to the expression of act-1. At least 3000 *C. elegans* were used in each group and the experiments were performed on three independent occasions. Means with different letters were significantly different at *P* < 0.05 for each gene. (**B**) RMF extended lifespan of the *hsf-1* (AGD794) and *eat-2* (DA1116) mutants. RMF did not extend lifespan of the *daf-2* (CB1370) and *daf-16* (CR1307) deletion mutants. The lifespan analysis of each mutant was performed on three independent occasions. (**C**) A possible mode of action of the longevity extension mediated by RMF.

Many studies have shown that insulin/igf-1 signaling pathway (IIS) is involved in regulation of biological process such as the life cycle in *C. elegans*. After insulin-like peptide ligands bind to the insulin/igf-1 transmembrane receptor (IGFR)-derived daf-2, daf-2 ultimately regulates FOXO transcription by controlling the phosphoinositide 3-kinase (PI3K)/Akt kinase cascade. Factor daf-16, then controls the life cycle and neurodegeneration process. To determine the role of the IIS pathway in the mechanism by which RMF increases longevity, we investigated the ability of RMF to promote *daf-16* transcription and translation. Under high-glucose conditions, 2 h/d RMF significantly increased daf-16 protein levels in *C. elegans*. In addition, real-time quantitative PCR analysis showed that RMF increased *daf-16* mRNA levels in *C. elegans* (Figure 10A–10C. *P*<0.01). Considering the antioxidant effect of RMF on *C. elegans*, we subsequently investigated the influence of RMF on SOD-3 (a downstream antioxidant gene in the IIS pathway). Quantitative real-time PCR analysis showed that SOD-3 mRNA levels were significantly increased in *C. elegans* following RMF exposure. Furthermore, evaluation of SOD-3 antioxidant enzyme protein levels in transgenic SOD-3::GFP (CF1553) revealed that SOD-3::GFP were increased following RMF treatment compared with the levels in the control group (Figure 10D–10F. *P*<0.01). Subsequently, we further investigated the involvement of other age-related genes (*age-1*, *ctl-1*, *sir-2.1*, *hsp-16.2*, *hsp-16.1*, *skn-1*, *gst-4*, and *gcs-1*) in the mechanism by which RMF increases longevity. We found that mRNA levels of *sir2.1*, *ctl-1*, *hsp-16.1*, *hsp-16.2* and *gcs-1* were significantly increased after RMF treatment. These results indicated that the effect of RMF on longer life span and increased stress resistance depends in part on the promotion of related stress-inducing genes (Figure 11A). Hsf-1 is a heat shock transcription factor known to play an important role in regulation of the insulin/igf-1 signaling pathway (IIS). According to previous reports, hsf-1 can downregulate the IIS pathway to further extend lifespan. The eat-2 gene plays a role in regulating the post-synaptic pumping rate of the pharyngeal muscle and is closely related to the DR? pathway. In this study, we evaluated the influence of RMF on the lifespan of *daf-2* (CB1370), *daf-16* (GR1307), *hsf-1* RMF-1 (AGD794) and *eat-2* (DA1116) mutants. The results show that hsf-1 is not required for the function of RMF and the DR pathway is not involved (*P*>0.05). This is consistent with the results of the food-intake experiment, indicating that RMF extends lifespan via the IIS pathway (Figure 11B, Table 3). Finally, we summarize the molecular mechanisms by which RMF interacts with HUVEC and infer the RMF action site and the magnetic induction pathway (Figure 11C).

**Table 3:** Statistical analysis of the lifespan of transgenic *C. elegans*.

**Condition**	**Treatment**	**Mean lifespan**	**Median lifespan**	**Maximum lifespan**	**Mean fold change**
	Control	35.8±0.47	34±1.64	50	—
CB1370	RMF 2h/d	36.0±0.34	36±0.29	54	0.56%
(15°C, days)	Glucose	34.8±0.96	38±0.93	48	-2.79%
	Glucose RMF 2h/d	34.3±1.33	34±0.86	48	-4.19%
	Control	17.2±0.18	20±0.73	22	—
GR1307	RMF 2h/d	18.9±0.64	20±0.81	24	9.88%
(20°C, days)	Glucose	16.3±0.34	18±0.34	22	-5.23%
	Glucose RMF 2h/d	16.1±0.18	18±1.39	22	-6.40%
	Control	16.3±0.14	18±0.17	22	—
AGD794	RMF 2h/d	16.7±0.62	18±0.14	26	2.45%
(20°C, days)	Glucose	12.7±0.97	13±1.13	18	-22.09%
	Glucose RMF 2h/d	14.5±0.39	16±0.91	22	-13.17%
	Control	35.1±0.21	36±0.73	50	—
DA1116	RMF 2h/d	35.4±1.34	37±0.91	54	0.85%
(20°C, days)	Glucose	25.1±1.02	26±0.55	40	-28.49%
	Glucose RMF 2h/d	25.7±0.71	24±0.57	42	-26.78%

The data were analyzed by one-way ANOVA (SPSS 18), and different letters in a column denote values that are significantly different (*P* < 0.05). The mean fold increase was calculated by (T-C)/C×100, where T is the mean survival time of transgenic *C. elegans* treated with RMF and C is the mean survive time of the control.

## DISCUSSION

Numerous studies in humans and *in vivo* models have shown that magnetic fields have multiple biological effects. Magnetic fields can be used to regulate biological magnetic fields in the body, by producing small currents that cause changes in cell membrane permeability, alter the activity of some enzymes, shrink blood vessels and accelerate blood flow. Magnetic fields have also been used to achieve ancillary treatment effects, such as pain relief and reduction of swelling. Mercado-Sáenz S et al. previously reported that (PMF) exposure resulted in an acceleration of cellular aging in *Saccharomyces cerevisiae*, which was not observed in the group treated with a sinusoidal magnetic field (SMF) [56]. The results of our study indicate that RMF with a frequency of 4 Hz and an intensity of 0.2 T delays the HUVEC senescence and prolongs the lifespan of *C. elegans*. Compared with SMF, the direction of the magnetic field in RMF is constantly changing, and it will generate an electronic eddy current at the site affected by the magnetic field. In a single-direction magnetic field, proteins that are affected by a magnetic field change structure in only one direction, although the effects of electronic eddy currents on the spatial structure of the protein will be greater and the range of action will be greater. However, its specific mechanism is still unclear. In addition, we found that the effect of RMF exposure on inhibition of aging depends on frequency, intensity, and duration of action. Sensitivity to the induced magnetic field and/or current in the animal changes during aging.

It is generally believed that very low strength MF has a certain effect on suppressing aging. Our research shows that RMF with the lowest intensity (0.2T) delays aging. We previously reported a similar promotion of cell proliferation at low intensity RMF [57, 58]. Based on our long-term research, we believe that the biological effects of RMF are related to the synchronous motion of charged particles in target tissues/cells. Taking a cell as an example, when the Larmor radius of the RMF is larger than the radius of the target cell, the charged particles in the whole cell move synchronously toward the center; otherwise, the synchronous motion of the charged particles is locally limited. The motion of charged particles is irregular overall. To the best of our knowledge, there have been no reports of RMF-induced aging. The relationship between ion channels and aging, and the effects of magnetic fields on ion channels have been extensively reported. Our study focused on finding direct and indirect sites of action through which RMF delays aging. We investigated the stimulatory effects of RMF exposure at different times, focusing specifically on the regulation of Ca^2+^ concentration by RMF. We adopted this approach based on reports that magnetic field exposure opens up the Na^+^ channels on the cell membrane, leading to increased Na^+^ levels that in turn, increase the intracellular Ca^2+^ concentration and the production of intracellular microtubules [59]. However, in this study, we found that the RMF acts directly act on the calcium channel of the endoplasmic reticulum, possibly due to the greater influence of the RMF compared with the SMF, which amplifies the effect of the magnetic field.

In recent studies, life-extending drugs or other interventions have not necessarily slowed the aging process; however, delayed aging will not only increase life expectancy, but as the population ages, will have a profound impact on the global population.

Glucose stimulates HUVEC-induced senescence, while RMF delays high-glucose-induced HUVEC senescence to some extent via the Ca^2+/^AMPK signaling pathway. We evaluated the ability of RMF to allow endoplasmic reticulum Ca^2+^ to flow into the cytoplasm to reduce the MMP and ATP content, activate the AMPK pathway, and inhibit the mTOR pathway. In animal studies, the RMF significantly increased the pharyngeal pump and body movement index of N2 *C. elegans*, and significantly reduced the accumulation of fat and lipofuscin, but had no influence on feeding and reproduction in *C. elegans*. RMF significantly increased the resistance of nematodes to adverse conditions such as heat stress and effectively reduced ROS levels, indicating that RMF promoted longevity. We also found that RMF exposure reduced the ATP level of the entire nematode, which was consistent with the effects observed in HUVECs.

Based on the results of our HUVEC and *C. elegans* studies, we examined mRNA and protein levels of daf-16 and sod-3, which are key factors in the IIS pathway, suggesting that this pathway is involved in the RMF-induced extension of lifespan. Furthermore, we observed that RMF significantly upregulated the expression of the stress genes sir2.1, ctl-1, hsp-16.1, hsp-16.2 and gcs-1. Subsequent analysis showed that the ability of RMF to extend lifespan in *C. elegans* is mediated via the IIS and anti-stress pathways, independent of the DR pathway. Since many studies have shown that autophagy plays an important role in aging and protein homeostasis, we propose that RMF increases autophagy to prolong life. In this study, RMF delayed HUVEC senescence and prolonged the healthy lifespan of *C. elegans* via the Ca^2+/^AMPK/IIS stress and autophagy pathways. These pathways are highly conserved from *C. elegans* to mammals, and our findings may help promote healthy aging and the use of RMF to treat age-related diseases in humans.

Recent studies have shown that the vast majority of research groups focus on the increase in Ca^2+^ concentration in response to intracellular endoplasmic reticulum stress, which can cause apoptosis or even premature aging [60, 61]. However, our results show that an appropriate increase in intracellular Ca^2+^ concentration is beneficial in slowing the rate of intracellular metabolism, which may explain the longevity of certain organisms, such as turtles. Strong magnetic fields tend to damage normal biological functions ranging from growth to mental awareness. However, it cannot be ignored that our daily life is constantly affected by the magnetic field of the earth as a natural magnet. The influence of weak magnetic fields on life is rarely studied in this context. Our research group focuses on finding the optimal magnetic field conditions to promote life. Thus, our experimental results can be used as a reference for future research.

## MATERIALS AND METHODS

### RMF system

RMFs were generated by the Rotating Magnetic Bed System (RMBS; designed by Prof. Xiaoyun Zhang University of Shenzhen, China), equipped with a permanent magnet capable of generating up to 0.2 T RMF. The RMBS was equipped with a closed glass chamber, which included a heating element and a gas-holding device to maintain a defined environment for *C. elegans* and cells. *C. elegans* were maintained according to standard protocols.

### C. elegans

The following strains were used in this study: the wild-type N2, Bristol (wild-type); GR1307, daf-16 (mgDf50); CB1370, daf-2 (e1368); TK22, mev-1 (kn1); AGD794, hsf-1; DA1116, eat-2; DLM1, dFP:: LGG-1; TJ356 [zIs356IV(daf16p::daf16a/b::GFP+rol6 (su1006))]; CF1553 [(pAD76)SOD-3::GFP+rol6 (su1006)]; and CL4176 [(pAF29) myo-3p::Aβ1-42+(pRF4)rol6(su1006)].

### Cell culture

HUVECs from neonatal umbilical cord were washed three times with phosphate-buffered saline (PBS) and digested with 0.2% type II collagenase at room temperature for 15 min. After centrifugation for at 1,200 r/min for 5 min, cells were resuspended in complete medium in the process of the temperature after abandon supernatant (800 mL/L M199, 200 mL/L FBS, 20 μg/mL ECGS, 1 mmol/L glutamine) heavy suspension cells, beat after blending in cultivation in the bottle. Passage 1–2 cells were used for follow-up experiments.

### Lifespan measurement

Lifespan was measured in 3.5 cm tissue culture plates containing 10 μM 5-fluoro-2′-deoxyuridine (FUDR). *C. elegans* at the young adult stage were cleaned and collected for disruption with 5% sodium hypochlorite solution (composed of 0.4g NaOH, 4mL H2O, 2mL 10% bleach), and cleaned with M9 (1LH2O, 3gKH2PO4, 6gNa2HPO4, 5gNaCl and 1ml 1molLMgSO4) solution three times. The eggs and M9 were then transferred to the 3.5 cm tissue culture plates for synchronization. The synchronized *C. elegans* were grown to the adult stage on the NGM (2.5g peptone, 3.0g NaCl, 17g agar powder and 975ml water were sterilized by high temperature and cooled to 55 degrees Celsius. Stirred CaCl2, MgSO4, cholesterol solution and PBS were shaken to the plate) plate inoculated with *Escherichia coli* OP50, and were then were transferred to plates containing *E. coli* OP50 alone, and maintained at 20°C as described previously [62]. Subsequently, *C. elegans* were transferred to a new petri dish every 2–3 days until no response to mechanical stimulation was observed, when the nematodes were presumed dead. The significance of differences in the survival curves were evaluated by log-rank tests. There were more than 100 *C. elegans* in each group, and each experiment was repeated on at least three independent occasions. Lifespan tests were carried out 20°C, and heat shock experiments were carried out at 37°C.

### Healthspan evaluation

The responses of nematodes to radiation and physical stimuli were evaluated every two days and death was determined by the lack of response. To measure head-swing and body-bending frequencies as well as locomotivity, *C. elegans* maintained on NGM plates without food were treated with or without RMF 2 h/d for 6, 10, and 14 d. This assay was repeated on two independent occasions. Each group included at least 30 *C. elegans* [63, 64].

### Brood size

The incubation size of an adult worm was measured. Starting from the L1 stage, *C. elegans* were first treated with RMF for 2 h/d, and then maintained on NGM plates with *E. coli* OP50 at 20°C for 72 h to allows each worm to lay eggs. The eggs were then allowed to hatch and counted. The experiment was repeated on three independent occasions.

### Measurement of ROS accumulation

Endogenous ROS levels were measured using 2′,7′-dichlorofluorescein diacetate (H2DCF–DA). On day 10 after RMF2 h / d treatment, one hundred *C. elegans* were incubated with 10 μM H2DCF-DA for 30 μ min at 37 °C. Fluorescence intensity (excitation and emission wavelengths of 485 and 535 nm, respectively) was measured. The assay was performed on three independent occasions.

### Measurement of fat accumulation

To investigate the effect of 2 h / d RMF treatment on fat accumulation, RMF was treated for 7 days, 10 days, and 14 days, followed by oil red O staining. The *C. elegans* were then immobilized under a fluorescence microscope and visualized with paraformaldehyde. The fat accumulation test was repeated three times. ImageJ software (National Institutes of Health, Bethesda, MD, USA) was used to analyze the images and calculate the total number of *C. elegans* (at least 60 in each group).

### Analysis of cytosolic Ca^2+^

HUVECs were seeded in culture dishes (35 mm thin-bottomed and 7 mm low wall; Ibidi, #80136, Germany) and cultured for three generations. HUVECs were loaded with 2 mM Fluo-4-AM (Solarbio, F8500, China), cell clear fluorescent Ca^2+^ probe and 0.04% pluronic F-127 in HEPES-buffered Tyrode solution (HEPES 10 mM, KCl 2.8 mM, NaCl 129 mM, MgCl_2_ 2 0.8 mM, NaHCO 3 8.9). M1, KH_2_PO_4_ 0.8 mM, glucose 5.6 mM, CaCl_2_ 1.2 mM and 0.1% BSA, pH 7.4 were incubated at 37°C as described previously [65]. Then, the myotubes were washed with buffer and incubated for an additional 40 min at room temperature. The acetoxymethyl ester (AM) group was cleaved by intracellular esterase. The endoplasmic reticulum calcium channel inhibitor BAPTA (MCE, HY-100168, China) was added during the AM cutting phase. HUVECs were then analyzed at 37°C. Cells were placed 6 cm above the magnetic coil and exposed to RMF using a 20 min kinetic cycle. Three RMFs were applied during this cycle: 0–4 min (interval 1), 8–12 min (interval 2) and 16–20 min (interval 3); these three intervals were marked as “ON”. In between, the RMF was switched off for 4 min; this interval was marked as “OFF”. Changes in cytosolic Ca^2+^ were monitored using an automated inverse fluorescence microscope (Ti-E, Nikon, Japan) and flow cytometry (BD C6, USA) at an excitation wavelength (Ex) of 490 nm and an emission wavelength (Em) of 515 nm.

### Mitochondrial membrane potential detection

Changes in MMP were evaluated using a MMP detection kit (JC-1) (Solarbio, M8650). HUVECs were exposed to RMF for 0 h, 4 h, 4 h plus BAPTA. After treatment, the medium was removed and the cells were washed once with PBS before adding fresh medium and JC-1. Cells were incubated at 37°C for 20 min before the JC-1 staining buffer was removed by washing twice. The cells were digested and analyzed by flow cytometry.

### Total ATP detection

The ATP content of *C. elegans* and HUVECs was analyzed using an ATP Analysis System Bioluminescence Detection Kit (Promega, FF2000). HUVECs (20,000/well) or nematodes (20/well) were added to 96-well plates. The ATP kit test solution was added and plates were incubated for 1 h at room temperature. The ATP content was detected by chemiluminescence.

### RNA sequencing

Gene expression in HUVECs treated with RMF for 4 h or the vehicle control was performed on day 10 with the assistance of Novo Gene Corporation (Beijing, China). Up- or down-regulated genes were identified by filtering the RNA-seq data with the following cut-off: twofold change in expression level and a false discovery rate analogue of q value less than 0.05.

### Quantitative real-time PCR (RT-PCR)

Approximately 500 synchronized C*. elegans* were cultured at 20°C with or without RMF 2 h/d. Total RNA was extracted using TRIzol A+ (Tiangen, China). RNA (3 μg) was reverse transcribed using a GoScript Reverse Transcription System (Promega, A5004) as described previously. The expressed genes were amplified in triplicate and quantified in a SYBR Green PCR Mix (Applied Biosystems) with an ABI 7500 DNA Analyzer (Applied Biosystems) as described previously [66]. Each experiment was performed in triplicate and the fold change was calculated by a relative quantification method (2-∆CT). The primer sequence of the gene to be detected is in Supplementary Table 1.

### Western blot analysis

After treatment, the culture medium was discarded, and cells were washed twice with precooled PBS. Appropriate amounts of cell lysate were added, and cell protein was scraped with a cell blade after the ice was fully cleated. The supernatant was a centrifuged at 12,000r/min for 10 min at 4°C for, and the protein content was determined using the BCA method. Proteins were boiled for 5–10 min and separated by SDS-PAGE before transfer to a PVDF membrane. Membranes were then blocked with 5% skimmed milk powder solution at room temperature for 1 h, and then incubated overnight at 4°C with primary antibodies for the detection of P21, P53, AMPK (Invitrogen, MA5-12557, MA5-15815, all 1:1,000) and mTOR (CST, #2983, 1:1,000). After washing three times with 1× TBST for 5 min, membranes were incubated with the appropriate secondary detection antibody (Invitrogen, G-21040, G-21234, all 1:10,000) at room temperature for 1 h. After washing three times with 1× TBST for 5 min, the antibody-reactive protein bands were detected by chemiluminescence.

### Statistical analysis

All statistical analyses were conducted with GraphPad Prism 8.0. Lifespan and paralysis assay data were analyzed using the Kaplan–Meier survival method and *P*-values were calculated using the log-rank test. Student’s *t*-test was used to analyze differences between two groups. One-way analysis of variance (ANOVA) with Duncan’s test was used to compare multiple groups. *P* < 0.05 was considered to indicate statistical significance.

## Supplementary Material

Supplementary Table 1
