# Supplementary material for: Rotating magnetic field delays human umbilical vein endothelial cell aging and prolongs the lifespan of Caenorhabditis elegans
Source: Aging (Albany NY). 2019 Nov 22;11(22):10385–408. doi: 10.18632/aging.102466 (PMC6914427; doi:10.18632/aging.102466)
Supplement: Supplementary Table 1 [file aging-11-102466-s001..pdf]

SUPPLEMENTARY TABLE

Supplementary Table 1. The primer sequence of the gene.

atg-18 F	AAA TGG ACA TCG GCT CTT TG
atg-18 R	TGA TAG CAT CGA ACC ATC CA
lgg-1F	acc cag acc gta ttc cag tg
lgg-1R	acg aag ttg gat gcg ttt tc
sqst-1F	TGG CTG CTG CAT CAT CCG CT
sqst-1R	TCA ATC GTG CCG AGA CCG GG
vps-11F	TCC GCT TGT CGT CCT GGA GC
vps-11R	TCA CAC GCC GAG CAC TTG GT
vha-15F	CGA GGT TCG TTC CGG ACG TCT T
vha-15R	CCT CGG CAG TCA GGA GAC GC
vha-16F	AGG CGC TGA CTC GCG GAC TT
vha-16R	TGG TCT CTG GTG AAG AGT TCC GGT G
act-1F	TCCTTACCGAGCGTGGTTAC
act-1R	GTTTCCGACGGTGATGACTT
atg-18F	CAGAAGCGTCTACCGAAGGT
atg-18R	GATTACTCCTTGCGGCTCCT
bec-1F	TTCGTCGATTCTTGCAGTTG
bec-1R	TCTGAGAGCATCGCATTGAG
mtl-1F	TGAGGAGGCCAGTGAGAAAAA
mtl-1R	GCTCTGCACAATGACAGTTTGC
ftn-1F	TGACGCGCACTTGACAAATTA
ftn-1R	TGTAGCGAGCAAATTCATTGATC
dod-6F	CTCAAGACCGTCGCCCTCTA
dod-6R	TCAGCATCAGCGCAAGCA
lys-7F	CATTCGGCATCAGTCAAGGTT
lys-7R	GCAGGCTCCGCAATGACTT
hsp-16.2F	TACGCTATCAATCCAAGGAGAAC
hsp-16.2R	GAAGCAACTGCACCAACATC
pmp-3F	GAATGGAATTGTTTCACGGAATGC
pmp-3R	CTCTTCGTGAAGTTCCATAACACGATG
gpd —1F	TCAAGGAGGAGCCAAGAAGG
gpd —1R	CAGTGGTGCCAGACAGTTG
age —1F	CCTGAACCGACTGCCAATC
age —1R	GTGCTTGACGAGATATGTGTATTG
daf —2F	GCGGATACACAGCAAGAATAAC
daf —2R	GAGCCACAAGCACCAGAAC
sir —2.1F	ACTGAGATGCTCCATGACAATAAG
sir —2.1R	GCAAGACGAACCACACGAAC
sod —3F	GGCTAAGGATGGTGGAGAAC
sod —3R	ACAGGTGGCGATCTTCAAG
daf —16F	TCAAGCCAATGCCACTACC
daf —16R	TGGAAGAGCCGATGAAGAAG
ctl —1F	CGGATACCGTACTCGTGATGAT
ctl —1R	CCAAACAGCCACCCAAATCA
ama-1F	TGG AAC TCT GGA GTC ACA CC
ama-1R	CAT CCT CCT TCA TTG AAC GG
nhr-23F	CAG AAA CAC TGA AGA ACG CG

nhr-23R	CGA TCT GCA GTG AAT AGC TC
akt-1 f	5′-TCACCGATGCGATTGTCT-3′
akt-1 r	5′-AACTCCCCACCAATCAACAC-3′
skn-1 f	5′-AGTGTCGGCGTTCCAGATTTC-3′
skn-1 r	5′-GTCGACGAATTGCGAATCA-3′
gsh-px f	5′- ATGGCACTTTGGCAGCTCA-3′
gsh-px r	5′- ACGCGCAAAAAGTAGCAACGC-3′
gst-4 f	5′- ATGCTCGTGCTCTTGCTGAG-3′
gst-4 r	5′- GACTGACCGAATTGTTCTCCAT-3′
gcs-1 f	5′- GTCGATGAAGCCAGATGGTTGT-3′
gcs-1 r	5′- CGATCGTCGACACTTGCACTAA-3′
